# Why is immunotherapy for glioblastoma “Lag”-ging

**DOI:** 10.18632/oncotarget.26648

**Published:** 2019-02-08

**Authors:** Dimitrios Mathios, Michael Lim

**Affiliations:** Department of Neurosurgery, Oncology and Radiation Oncology, Johns Hopkins Hospital, Baltimore, MD, USA

**Keywords:** LAG-3, PD-1, glioblastoma

Immune checkpoint inhibition for solid tumors has revolutionized treatment outcomes the last several years. This class of immunotherapeutic agents works by preventing or reversing exhaustion of tumor specific immune effector T cells. Despite the original success of these agents with some solid tumors [1-3], glioblastoma has been notoriously unresponsive to immune checkpoint inhibition [4].

There is a variety of factors that this lack of treatment response has been attributed to, including the blood brain barrier, the lack of immunogenicity of glioblastoma, or the low levels of expression of PD-1 or PD-L1 compared to other tumor types. An additional reason to the lack of therapeutic benefit of anti-PD-1 mAbs is the fact that other immune checkpoints can be upregulated during the course of disease progression and despite blocking one (i.e. PD-1/PD-L1) pathway others can be upregulated. Immune checkpoint molecules, like CTLA4, LAG3, TIM3, GITR can be upregulated during the course of glioblastoma progression and can effectively work as resistance mechanisms to anti-PD-1 treatment.

LAG3 has specifically attracted attention lately as it has shown significant preclinical efficacy in many tumor models in combination with anti-PD-1 [5, 6, 7]. The importance of LAG-3 in infectious disease (especially in HIV research as a marker of T cell exhaustion) has been well established many years before its implication in cancer. LAG-3 is expressed on CD4 cells, promoting maturation of T suppressor cells. More lately soluble LAG-3 has been found to lead to maturation of plasmacytoid dendritic cells that suppress immune responses [8].

In glioblastoma specifically, our group published preclinical data recently on the efficacy of the combination of anti-PD-1 and anti-LAG-3 mAbs [9]. Functional analysis of the tumor infiltrating lymphocytes showed that CD4 or CD8 cells that express PD-1 and not LAG-3 are highly functional T cells that express high levels of IFN-γ. Conversely, CD4 or CD8 cells expressing LAG-3 and not anti-PD-1 exhibited an exhaustive phenotype with very low levels of IFN-γ production. These results imply that LAG-3 is a marker of T cell exhaustion. Additionally, our model showed that the sooner the treatment with anti-LAG-3 mAb starts, the more efficacious the treatment is in prolonging survival. We postulate this is the case, as the earlier LAG-3 is blocked the faster T cell dysfunction can be reversed. However, it is unclear what molecular processes are regulating reversal of T cell dysfunction leading to T cell exhaustion, after surface expression of LAG-3; more important, the timeline of LAG3 inhibition in relation to T cell exhaustion is unknown. Future studies elucidating this timeline and molecular mechanisms of T cell exhaustion in the setting of different cancer subtypes as well as optimal timing of administration of LAG-3 blocking antibody are crucial.

In terms of available clinical data from use of LAG-3 inhibition in cancer, exciting preliminary results have been published in advanced melanoma [10]; LAG-3 positive melanoma patients refractory to anti-PD-1 treatment treated with anti-LAG-3 mAb in combination with anti-PD-1 mAb exhibited a >3 fold increase in objective treatment response rate (18% *vs* 5%) compared to LAG-3 negative tumor patients. Our group and others have started clinical trials in a variety of solid and hematological malignancies combining anti-LAG-3 with other immune checkpoint inhibitors.

As more patients receive treatment with anti-LAG-3 and anti-PD-1 mAbs clinicians will have to be vigilant of the adverse events that may come with the combination of these two antibodies. PD-1/LAG-3 knock out mice are short lived (<4 weeks) due to lethal autoimmune endocarditis or pancreatitis. As with other immunecheckpoint inhibitors the combination of anti-LAG-3 with other therapies have to be examined critically not only of the survival outcome but also of the potentially dangerous autoimmune sided effects of these treatments. For this reason it is important to identify biomarkers of response to the treatment with anti-LAG-3 mAb and identify the subgroup of patients that will benefit most.
